# Functional near-infrared spectroscopy in movement science: a systematic review on cortical activity in postural and walking tasks

**DOI:** 10.1117/1.NPh.4.4.041403

**Published:** 2017-08-01

**Authors:** Fabian Herold, Patrick Wiegel, Felix Scholkmann, Angelina Thiers, Dennis Hamacher, Lutz Schega

**Affiliations:** a Otto von Guericke University Magdeburg, Institute III, Department of Sport Science, Magdeburg, Germany; b University of Freiburg, Department of Sport Science, Freiburg, Germany; c University of Zurich, University Hospital Zurich, Department of Neonatology, Biomedical Optics Research Laboratory, Zurich, Switzerland

**Keywords:** functional near-infrared spectroscopy, optical neuroimaging, motor control, walking, posture

## Abstract

Safe locomotion is a crucial aspect of human daily living that requires well-functioning motor control processes. The human neuromotor control of daily activities such as walking relies on the complex interaction of subcortical and cortical areas. Technical developments in neuroimaging systems allow the quantification of cortical activation during the execution of motor tasks. Functional near-infrared spectroscopy (fNIRS) seems to be a promising tool to monitor motor control processes in cortical areas in freely moving subjects. However, so far, there is no established standardized protocol regarding the application and data processing of fNIRS signals that limits the comparability among studies. Hence, this systematic review aimed to summarize the current knowledge about application and data processing in fNIRS studies dealing with walking or postural tasks. Fifty-six articles of an initial yield of 1420 publications were reviewed and information about methodology, data processing, and findings were extracted. Based on our results, we outline the recommendations with respect to the design and data processing of fNIRS studies. Future perspectives of measuring fNIRS signals in movement science are discussed.

## 1 Introduction

Safe locomotion is indispensable for human daily living and requires good functionality of motor control processes. The efficiency of motor control processes of daily motor activities such as walking^1^^,^^2^ and standing^3^^,^^4^ relies on complex neuronal networks encompassing subcortical and cortical brain structures. Studies show that a smaller gray matter volume is associated with lower gait performance indicated by increased gait variability^5^^–^^7^ or slower gait velocity.^8^^,^^9^ Moreover, lower whole-brain gray matter volume goes along with worse postural balance performance irrespective of age,^10^ whereas the increase of gray matter volume is associated with balance improvements.^11^^–^^13^ In older age, however, shrinking of those cortical structures^14^^,^^15^ might diminish motor control capabilities.^16^ The substantial body of literature suggests that cortical structures play an important role for the motor control of daily motor tasks. Therefore, the assessment of cortical activity while subjects are moving is a key factor to foster a better understanding of neuromotor control which, in turn, could help to improve rehabilitation strategies.^17^

Brain activity can be measured by the following neuroimaging methods: functional magnetic resonance imaging (fMRI), magnetoencephalography (MEG), positron-emission-tomography (PET), electroencephalography (EEG), and functional near-infrared spectroscopy (fNIRS). While fMRI is considered as gold standard for the assessment of activity in cortical and subcortical structures, it suffers from the vulnerability for movement artifacts and the restricted range of motion in the scanner.^18^^–^^21^ Likewise, MEG exhibits a high vulnerability for motion artifacts^18^ while the use of PET does not allow repeated measurements due to the injection of radioactive tracers.^20^ EEG puts out not only signals with high temporal resolution but also signals with a relatively weak spatial resolution.^18^^,^^22^ Furthermore, EEG is vulnerable to artifacts, time consuming in preparation,^18^^,^^22^^,^^23^ and the signals are hard to interpret for nonexperts.^24^ Hence, fMRI, MEG, PET, and EEG suffer from specific restrictions that hamper a time-efficient evaluation of cortical activation in moving subjects.

fNIRS is a relatively new optical neuroimaging technique that uses the theory of neurovascular coupling.^19^^,^^25^^–^^27^ Neurovascular coupling results from the neuronal activity or glia activity that provokes an enhanced blood flow in an active brain region to satisfy energetic demands of the neuronal tissue.^27^^–^^29^ Based on these hemodynamic responses of neuronal cortical tissues, the fNIRS technology allows an indirect evaluation of brain activation (such as fMRI).^18^^,^^19^

Therefore, light with different wavelengths in the near-infrared spectrum is emitted through the skull and undergoes some scattering and absorption processes inside the neuronal tissue.^27^^,^^30^^,^^31^ In the neuronal tissue, the chromophores such as oxygenated (oxyHb) and deoxygenated hemoglobin (deoxyHb) absorb light at different spectra^19^^,^^20^^,^^32^^,^^33^ whereas the nonabsorbed components of the scattered light leave the brain in a banana-shaped course. Those components are recorded by a detector on the head surface.^30^^,^^31^^,^^34^ Based on the described neurovascular coupling, an enhanced brain activation induces an intensified blood flow in the active brain regions leading to an increase in oxyHb and decrease of deoxyHb.^27^^,^^30^ As a consequence of the different absorption spectra of the chromophores, the activity-dependent concentration changes in oxy- and deoxyHb can be quantified with the modified Beer–Lambert law and used as an indicator of regional brain activation.^19^^,^^20^^,^^27^^,^^30^^,^^32^

The advantage of optical neuroimaging using fNIRS is the possibility to measure cortical activity (quantified as changes in tissue oxygenation and blood perfusion, associated with neural activity) noninvasively^25^^,^^27^^,^^35^^,^^36^ with a relatively good spatial and temporal resolution.^19^^–^^22^ This benefit makes fNIRS systems suitable for the usage in special cohorts, such as children.^18^^,^^20^^,^^22^^,^^36^^–^^40^ Moreover, fNIRS systems are applicable even during outdoor activities^41^ and could be used as a monitoring tool in neurorehabilitation settings.^18^^,^^42^^–^^44^ From this point of view, fNIRS is a promising tool to understand the contribution of cortical areas in the neuromotor control of gross motor skills, such as posture and walking.^17^ However, the fNIRS technology also has some disadvantageous including a limited depth sensitivity that restricts the measurements of brain activity to cortical layers^36^ and the vulnerability to systemic vascular changes that may contaminate the signal during strenuous physical tasks.^27^^,^^45^ In addition, no standardized procedures regarding the usage of fNIRS with respect to measuring cortical activity in moving subjects exist^17^^,^^42^ which clearly limits the comparability across existing studies.

This systematic review elucidates the application of fNIRS in neuromotor research and concentrates on two crucial motor tasks, namely locomotion and postural stability. In this context, we aim to give an overview about (a) the methodological approach of fNIRS and (b) the main findings of the fNIRS measurements reported in the literature.

## 2 Systematic Literature Search and Data Extraction

**Fig. 1:**
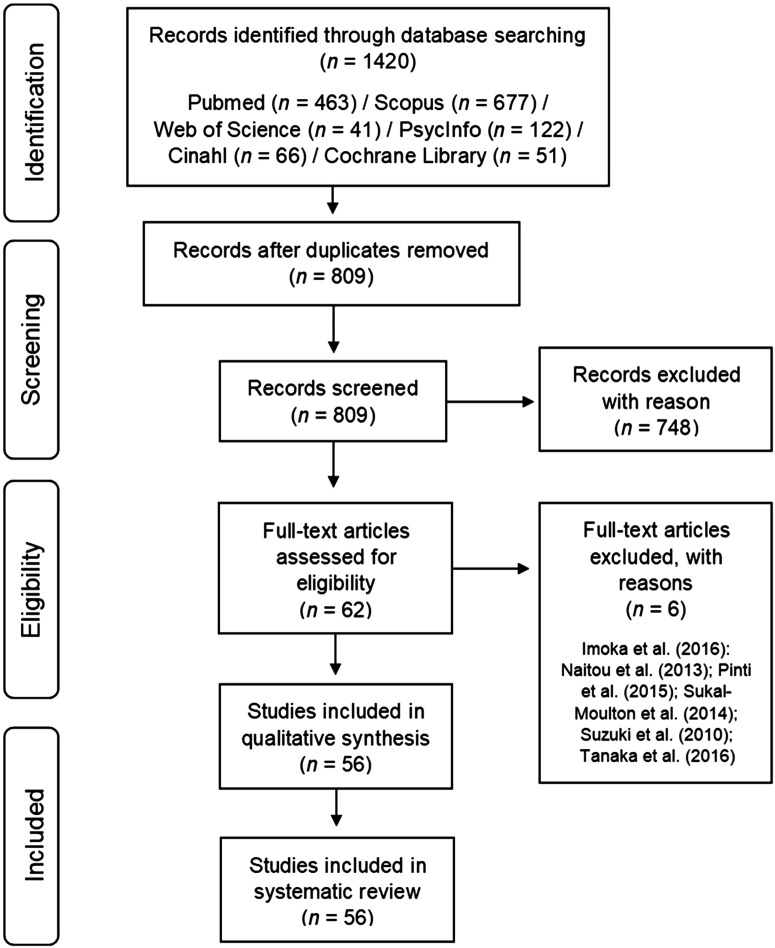
Search process and identification of relevant studies.

Two independent researchers performed a systematic literature search to identify all relevant studies applying fNIRS to investigate hemodynamic brain responses during walking and postural tasks on February 4, 2017. Therefore, we used the following search terms: gait OR walking OR posture OR “postural control” OR balance OR balancing OR sway AND NIRS OR fNIR OR fNIRS OR “functional near-infrared spectroscopy” OR “near-infrared spectroscopy” OR “functional near-infrared spectroscopic” OR “optical imaging system.” All studies that used brain–computer interfaces, examined orthostatic regulation or animals, provided insufficient statistical methods, or used non-English language were excluded. During this procedure, six articles were excluded due to the lack of statistical analyses,^46^^–^^48^ ineligible measurement condition,^49^ and non-English language.^50^^,^^51^ The search and screening process is shown in Fig. 1. From the included studies, data about cohort characteristics, fNIRS methodology, and main findings were extracted.

## 3 Results: Methodology Employed in the Studies

In the following, we will provide information about the methodological approaches of the reviewed studies. We focused on general aspects regarding the application, data processing, and data analyzing of fNIRS (e.g., study design, used filter methods, and statistical analysis). Further information about the cohorts, tasks, sampling frequencies, wavelengths, and numbers of channels can be requested by e-mail from the corresponding author or is available in Ref. 52.

### 3.1 Baseline Condition and Duration

#### 3.1.1 Treadmill walking

Fifteen studies investigating cortical activation during treadmill walking assessed baseline brain activation during quiet standing.^53^^–^^67^ In contrast, Eggenberger et al.^68^ chose slow walking (2  km/h) for 1 min as the baseline condition. The duration of baseline cortical activation used for further analyses varied between 2.5^55^^,^^56^ and 20 s^59^^–^^61^ (for an overview see Table 1).

**Table 1:** Overview about the population, study designs, and data processing steps of reviewed fNIRS studies (note that the number of trials is reported per condition).

			
First author	– Population (n=number of participants; age in years±SD)	1. Baseline condition	1. DPF
	• Conditions	2. Baseline duration	2. Data processing (filtering)
		3. Number of trials and duration	3. Final data processing
		4. Rest phase duration	4. Activation parameters
			5. Time used for analysis
Al-Yahya et al.^65^	– Stroke patients (n=19; 59.61±15.03)	1. Quiet standing	1. Age-dependent value ($4.99+0.067\times {\text{age}}^{0.814}$)
	– Healthy old adults (n=20; 54.35±9.38)	2. 25 to 45 s (randomized order)	2. LPF at 0.67 Hz
	• DTW vs. NW	3. 5×; 30 s	3. Baseline correction; averaging
		4. 25 to 45 s (between trials / randomized order)	4. Oxy- and deoxyHb
			5. 6 to 16 s after task begin
Atsummori et al.^69^	– Healthy young adults (n=6; 29.7±3.3)	1. Quiet standing	1. Constant value (no details reported)
	• DTW vs. NW	2. 5 s before task begin	2. Not reported
		3. 5× (DTW) / 6× (NW); 10 s	3. Baseline correction; averaging
		4. 20 s at beginning	4. Oxy- and deoxyHb
			5. 6 s after task begin/ending
Basso-Moro et al.^98^	– Healthy young adults (n=16; 29±4.8)	1. Quiet standing	1. Age-dependent value ($4.99+0.067\times {\text{age}}^{0.814}$)
	• Perturbations in semivirtual reality with increasing difficulty	2. Last 30 s (of 2 min)	2. LPF at 0.1 Hz
		3. 7×; 45 s	3. Averaging
		4. 2 min after block	4. Oxy- and deoxyHb
			5. Last 10 s of perturbation
Beurskens et al.^105^	– Healthy young adults (n=15; 24.5±3.3)	1. Sitting on chair	1. Constant value (6.0)
	– Healthy old adults (n=10; 71.0±3.8)	2. 30 s	2. HRF-filter; wavelet MDL detrending algorithm
	• DTW vs. NW	3. 2×; 30 s	3. Moving standard deviation and spline interpolation, baseline correction, canonical HRF
		4. Not reported	4. Oxy- and deoxyHb
			5. Entire task time
Caliandro et al.^70^	– Patients with ataxic gait (n=14; 27 to 71)	1. Quiet standing	1. Constant value (5.93)
	– Healthy controls (n=20; 32 to 65)	2. Last 10 s of standing	2. LPF at 0.1 Hz
	• Patients vs. HC	3. 1×; 10 m	3. Baseline correction; averaging
		4. Not relevant	4. OxyHb
			5. Entire task time expect of first 5 s
Caliandro et al.^71^	– Patients with ataxic gait (n=19; 31 to 70)	1. Quiet standing	1. Constant value (5.93)
	– Healthy controls (n=15; 36 to 73)	2. Last 10 s of standing	2. LPF at 0.1 Hz
	• Patients vs. HC	3. 2×; 10 m	3. Baseline correction; averaging
		4. 30 min between trials	4. OxyHb
			5. Entire task time expect of first 5 s
Clark et al.^66^	– Older persons with mobility and somatosensory deficits (n=14; 77.1±5.56)	1. Quiet standing	1. N/A
	• Walking in normal shoes vs. texture insoles vs. barefoot vs. DTW	2. 10 s immediately before task	2. No filter
		3. 5× walking laps with 18 m (overground); 60 to 120 s (treadmill)	3. Averaging
		4. 2 min after task	4. TOI
			5. Entire task phase
Clark et al.^86^	– Older adults with mild mobility deficits (n=16; 77.2±5.6)	1. Quiet standing	1. N/A
	• NW vs. DTW	2. 10 s immediately before task	2. No filter
		3. 5× walking laps with 18 m	3. Averaging
		4. 2 min after task	4. TOI
			5. 10 s before task begin (preparation phase) and in steady phase/transition phase excluded
Doi et al.^72^	– Adults with mild cognitive impairment (n=16; 75.4±7.2)	1. Quiet standing	1. N/A (arbitrary unit)
	• NW vs. DTW	2. 10 s before walking	2. LPF at 0.05 Hz; linear fitting on baseline data
		3. 3×; 20 s	3. Averaging
		4. 30 s between trials	4. OxyHb
			5. Entire task period
Eggenberger et al.^68^	– Healthy old adults (dancing: n=19; 72.8±5.9; balance: n=14; 77.8±7.4)	1. Walking at 2 km/h	1. N/A (absolute values)
	• Dancing vs. balancing (before and after intervention)	2. Middle 40 s (of 1 min)	2. 60 s moving average: motion artifact correction (oxyHb: >2.5 and <−2.5 μM/deoxyHb: >1.5 and <−1.5 μM excluded); visual inspection of data
		3. 8×; 30 s	3. Averaging
		4. 30 s between trials (walking at 2 km/h)	4. OxyHb
			5. 1 to 7 s = acceleration phase; 10 to 25 s = steady state walking phase; 26 to 34 s = deceleration phase; 35 to 46 s = drop phase
Ferrari et al.^99^	– Healthy, young adults (n=22; 26.5±4.0)	1. Quiet standing	1. Age-dependent value ($4.99+0.067\times {\text{age}}^{0.814}$)
	• Balancing in semivirtual reality	2. Last 30 s (of 2 min)	2. LPF at 0.1 Hz
		3. 2×; 9 min	3. Averaging
		4. 2 min after block	4. Oxy- and deoxyHb
			5. 30 s per task
Fraser et al.^63^	– Healthy young adults (n=19; 21.83±1.92)	1. Quiet standing	1. Constant value (no details reported)
	– Healthy old adults (n=14; 66.85±5.26)	2. 5 s	2. No filter
	• NW vs. single cognitive task vs. easy DTW vs. hard DTW	3. Walking: 2×; 2 min; single cognitive task: 4×; 75 s; DTW 4×; 2 min (for each dual-task condition)	3. Averaging
		4. 30 to 60 s between trials	4. Oxy- and deoxyHb
			5. Entire task period
Fujimoto et al.^102^	- Patients with subcortical stroke (n=20; 60.2±9.5)	1. Quiet standing	1. N/A (arbitrary unit)
	• Postural test before/after rehabilitation	2. Time before next perturbation (ERD)	2. HPF at 0.01 Hz; PCA
		3. 15×; 1 s	3. Two parameter gamma HRF
		4. 5 to 15 s between trials (randomized)	4. Oxy- and deoxyHb
			5. Around perturbations
Fujita et al.^101^	– Healthy, young adults (low span group: n=13; 24.0±3.1 / high span group: n=16; 22.5±3.6)	1. Quiet standing	1. N/A (arbitrary unit)
	• Single- and dual-task mono- or bipedal standing	2. 10 s	2. LPF at 0.5 Hz; HPF at 0.01 Hz; 5 s moving average
		3. 3×; 20 s	3. Baseline normalization, baseline correction, averaging
		4. 10 s between trials	4. OxyHb
			5. Entire task time
Harada et al.^53^	– Healthy, old adults (n=15; 63±4)	1. Quiet standing	1. N/A (arbitrary unit)
	• Low vs. high gait capacity group at different speeds	2. 10 s before walking	2. HPF at 0.03 Hz
		3. 3×; 60 s	3. Baseline normalization; averaging
		4. 40 s between trials	4. OxyHb
			5. 20 s after target speed
Helmich et al.^108^	– Young, concussed adults with persistent postconcussive symptoms (n=7; 29±15)	1. N/A	1. Constant value (6.0)
	– Young, concussed adults with minor postconcussive symptoms (n=13; 26±7)	2. N/A	2. LPF at 0.1 Hz; HPF at 0.001 Hz; spline interpolation; visual inspection
	– Healthy, young adults (n=10; 27±8)	3. 10×; 10 s	3. Normalization; averaging
	• Comparison of three groups during standing on different surfaces (stable vs. instable) and sensory conditions (eyes closed vs. eyes open vs. blurred vision)	4. No rest between trials	4. Oxy- and deoxyHb
			5. Entire task time
Hernandez et al.^82^	– Healthy old adults (n=8; 61±4)	1. Quiet standing	1. Constant value (6.0)
	– Patients with multiple sclerosis (n=8; 57±5)	2. 10 s before walking (counting silently in steps of 1)	2. LPF at 0.14 Hz; noisy channels excluded (dark current condition or saturation); visual inspected
	• Comparison of healthy adults and patients with multiple sclerosis during NW and DTW	3. 3× walking loops (= 6× straight walks a 14 ft.)	3. Baseline normalization; averaging
		4. At least 10 s after trial	4. OxyHb
			5. Entire task time
Herold et al.^100^	– Healthy young adults (n=10; 25; 21 to 47)	1. Quiet standing	1. N/A (arbitrary unit)
	• Standing vs. balancing on balance board	2. 30 s before task	2. 5.0 s moving average; LPF at 0.5 Hz; HPF at 0.01 Hz; PCA (r=0.25)
		3. 3×; 30 s	3. Averaging
		4. 30 s after trial	4. Oxy- and deoxyHb
			5. Middle 20 s
Holtzer et al.^73^	– Healthy, young adults (n=11; 19 to 29)	1. Quiet standing	1. Constant value (6.0)
	– Healthy, old adults (n=11; 69 to 88)	2. 5 s before walking	2. LPF at 0.14 Hz; combined principal and independent component analysis
	• DTW vs. NW vs. standing/comparison between cohorts	3. 3× walking loops (= 6× straight walks a 15 ft.)	3. Baseline normalization; averaging
		4. Not reported	4. OxyHb
			5. Old 4 s / young 3.5 s
Holtzer et al.^74^	– Nondemented older adults (n=318; 76.66±6.7)	1. Quiet standing	1. Constant value (6.0)
	• DTW vs. NW vs. standing	2. 10 s (counting silently forward in steps of 1)	2. LPF at 0.14 Hz; noisy channels excluded (dark current condition or saturation); visual inspected
		3. 3× walking loops (= 6× straight walks a 14 ft.) / standing for 30 s	3. Baseline normalization; averaging
		4. “Short break” reported	4. OxyHb
			5. Entire task time
Holtzer et al.^84^	– Nondemented older adults (n=348; 76.8±6.8)	1. Quiet standing	1. Constant value (6.0)
	– Older adults with low perceived stress (n=147; 76.72±6.87)	2. 10 s (counting silently forward in steps of 1)	2. LPF at 0.14 Hz; noisy channels excluded (dark current condition or saturation); visual inspected
	– Older adults high perceived stress (n=171; 76.58±6.37)	3. 3× walking loops (= 6× straight walks a 14 ft.) / standing for 30 s	3. Baseline normalization; averaging
	• DTW vs. NW vs. standing/comparison between cohorts	4. “Short break” reported	4. OxyHb
			5. Entire task time
Holtzer et al.^75^	– Nondemented older adults (total: n=236; 75.5±6.49)	1. Quiet standing	1. Constant value (6.0)
	– Healthy older adults (n=167; 74.43±6.04)	2. 10 s (counting silently forward in steps of 1)	2. LPF at 0.14 Hz; noisy channels excluded (dark current condition or saturation); visual inspected
	– Older adults with peripheral NGA (n=40; 77.03±6.27)	3. 3× walking loops (= 6× straight walks a 14 ft.) / standing for 30 s	3. Baseline normalization; averaging
	– Older adults with central NGA (n=29; 79.59±7.38)	4. “Short break” reported	4. OxyHb
	• DTW vs. NW vs. standing/comparison between cohorts		5. Entire task time
Holtzer et al.^85^	– Older adults with low perceived fatigue (n=160; 76.20±6.64)	1. Quiet standing	1. Constant value (6.0)
	– Older adults with high perceived fatigue (n=154; 77.41±6.66)	2. 10 s (counting silently forward in steps of 1)	2. LPF at 0.14 Hz; noisy channels excluded (dark current condition or saturation); visual inspected
	• DTW vs. NW vs. standing/comparison between cohorts	3. 3× walking loops (= 6× straight walks a 14 ft.) / standing for 30 s	3. Baseline normalization; averaging
		4. “Short break” reported	4. OxyHb
			5. Entire task time
Huppert et al.^91^	– Healthy young adults (n=10; 21 to 47)	1. Quiet standing	1. Not relevant (image reconstruction)
	• Stepping reaction task	2. Time before next trial (4 to 8 s, random order)	2. Discrete cosinus transform term (0−1/120 Hz); visual inspected
		3. 8× blocks a 32× trials	3. Gamma-variant HRF; averaging
		4. 4 to 8 s between trials (random order) / few minutes after 2 to 3 scans	4. Oxy- and deoxyHb
			5. Entire task phase
Karim et al.^97^	– Healthy young adults (n=9; 18 to 42)	1. Quiet standing	1. Not relevant (image reconstruction)
	• Video game with balance task	2. 60 s (pre- and posttask)	2. Cosinus transform term (0 to 1/120 Hz); visual inspected
		3. 6× beginner / 8× advanced level; 30 to 60 s	3. Boxcar HRF; averaging
		4. 30 s between trials	4. Oxy- and deoxyHb
			5. Entire task phase
Karim et al.^92^	– Healthy young adults (n=15; 28±9)	1. Quiet standing	1. Not relevant (image reconstruction)
	• SOT conditions	2. 45 s before trial	2. Cosinus transform term (0 to 1/120 Hz)
		3. 2×; 45 s	3. Gamma-variant HRF; averaging
		4. 60 s after trial / 2 min after two scans	4. Oxy- and deoxyHb
			5. Entire task phase
Kim et al.^106^	– Healthy young adults (n=14; 30.06±4.53)	1. Not reported	1. Not reported
	• Stepping (ST) vs. Treadmill walking (TW) vs. robot-assisted walking (RAW)	2. Not reported	2. Gaussian smoothing; wavelet MDL algorithm
		3. 5×; 30 s (ST, TW); 60 s (RAW)	3. Canonical HRF
		4. 15 s at begin and end; 30 s between trials (ST, TW) / 60 s at begin and end; 45 s between trials (RAW)	4. OxyHb
			5. Entire task time
Koenraadt et al.^54^	– Healthy, young adults (n=11/23±4)	1. Quiet standing	1. N/A (arbitrary unit)
	• Precision walking vs. NW	2. 25 to 35 s	2. LPF at 1.25 Hz; HPF at 0.01 Hz; superficial interference with LPF at 1 Hz; short separation channels (1 cm)
		3. 10×; 35 s	3. Baseline normalization; averaging
		4. 25 to 35 s before/after trial / 3 min after 10 trials	4. Oxy- and deoxyHb
			5. 12.5 s in task phase
Kurz et al.^55^	– Healthy, young adults (n=13; 23.7±1.4)	1. Quiet standing	1. N/A (arbitrary unit)
	• Forward vs. backward walking	2. 2.5 s before walking	2. HPF at 0.01 Hz; 5 s moving average; PCA (r=0.25)
		3. 10×; 30 s	3. Baseline correction; averaging
		4. 30 s between trials	4. Oxy- and deoxyHb
			5. Entire task phase
Kurz et al.^56^	– Children with spastic diplegic cerebral palsy (n=4; 11.0±4)	1. Quiet standing	1. N/A (arbitrary unit)
	– Healthy children (n=8; 13.2±3)	2. 2.5 s before walking	2. HPF at 0.01 Hz; 5 s moving average; PCA (r=0.25)
	• Patients vs. HC	3. 10×; 30 s	3. Baseline correction; averaging
		4. 30 s between trials	4. OxyHb
			5. Entire task phase
Lin et al.^103^	– Healthy middle-aged adults (n=15; 46±11)	1. Quiet standing	1. N/A (image reconstruction)
	– Healthy old adults (n=15; 73±5)	2. 40 s before trial	2. Autoregressive model with prewhitened iterative reweighted least squares algorithm
	• Middle-aged vs. old adults (different balance conditions)	3. 4×; 40 s	3. HRF; averaging
		4. 1 min between trials	4. Oxy- and deoxyHb
			5. Entire task phase
Lin and Lin^79^	– Healthy young adults (n=24; 20 to 27)	1. Quiet standing	1. Age-dependent value ($4.99+0.067\times {\text{age}}^{0.814}$)
	• DTW vs. NW	2. 20 s	2. LPF at 0.2 Hz
		3. 1×; 60 s	3. Baseline correction
		4. 20 s before/after task / 2 min after two trials	4. OxyHb
			5. Entire task phase
Lu et al.^76^	– Healthy young adults (n=17; 23.1±1.5)	1. Quiet standing	1. Constant value (6.0)
	• DTW vs. NW	2. 5 s before walking	2. Bandpass filter (LPF at 0.01 Hz; HPF at 0.2 Hz); PCA; spike rejection (channels with > CV 15% rejected/channels with CV > 10% for further analysis)
		3. 3×; 60 s	3. Averaging
		4. 60 s between trials	4. Hbdiff (oxyHb–deoxyHb)
			5. Early phase (5 to 20 s after task begin); late phase (21 to 50 s after task begin)
Mahoney et al.^93^	– Healthy, nondemented older adults (n=126; 74.41±6.12)	1. Quiet standing	1. Constant value (6.0)
	– Older adults wild mild Parkinson symptoms (n=117; 77.50±6.72)	2. First 2 s	2. LPF at 0.14 Hz; visual inspected
	– Patients with Parkinson disease (n=26; 81.23±5.93)	3. 10 s	3. Baseline normalization; averaging
	• Patients vs. HC (standing while counting silently in steps of 1)	4. “Short break” reported	4. OxyHb
			5. Entire task phase
Maidan et al.^90^	– Parkinson patients with FOG (n=11; 66.2±10.0)	1. Walking	1. Age-dependent value ($4.99+0.067\times {\text{age}}^{0.814}$)
	– Healthy controls (n=11; 71.2±6.0)	2. 6 s before FOG	2. LPF at 0.14 Hz
	• Patients vs. HC (walking; turning)	3. 6 s walking with 180 deg turn	3. Baseline correction; averaging
		4. 2 min between tasks	4. OxyHb
			5. Defined time period around FOG event (prior=−6 to −3 s / before=−3 to 0 / during=0 to 3 s)
Maidan et al.^80^	– Healthy, older adults (n=38; 70.4±0.9)	1. Quiet standing	1. Age-dependent value ($4.99+0.067\times \;{\text{age}}^{0.814}$)
	– Parkinson patients (n=68; 71.7±1.1)	2. 5 s before task	2. Bandpass filter (LPF at 0.01 Hz and HPF at 0.14 Hz), wavelet filter; CBSI
	• DTW vs. NW vs. obstacle negotiation	3. 5×; 30 s	3. Baseline correction; averaging
		4. 20 s after trial / between trials on individual needs	4. OxyHb
			5. Entire task phase
McKendrick et al.^88^	– Healthy, young adults (n=13; 22; 19 to 31)	1. Sitting (for sitting condition) and standing (for walking condition)	1. Constant value (5.94)
	• Sitting vs. walking indoors vs. walking outdoors (all conditions while performing secondary task)	2. 10 s	2. LPF at 0.1 Hz; visual inspected
		3. 16×; 120 s (sitting) / 8×; a 120 s (per walking condition)	3. Baseline correction
		4. 5 min between walking conditions	4. Oxy- and deoxyHb
			5. Entire task time
Meester et al.^57^	– Young, healthy adults (n=17; 27.8±6.3)	1. Quiet standing	1. Age-dependent value ($4.99+0.067\times {\text{age}}^{0.814}$)
	• DTW vs. NW	2. Middle 10 s of rest	2. LPF at 0.67 Hz; 4 s moving average; visual inspected
		3. 5×; 30 s	3. Averaging
		4. 20 to 40 s between trials	4. OxyHb
			5. Middle 10 s of task
Metzger et al.^64^	– Healthy young adults (n=12; 27.6; 19 to 39)	1. Quiet standing	1. N/A (arbitrary unit)
	• DTW vs. NW	2. 10 s at begin	2. 5 s moving average; CBSI
		3. 4×; 45 s	3. Averaging; baseline correction
		4. 15 s after trial	4. Oxy- and deoxyHb
			5. Entire task time
Mihara et al.^58^	– Stroke patients with ataxic gait (n=12; 52.7±16.9, 12 to 74)	1. Quiet standing	1. N/A (arbitrary unit)
	– Healthy controls (n=11; 42.6±11.6, 30 to 70)	2. 6 s before walking	2. Not reported
	• Patients vs. HC	3. 3×; 60 s (HC); 30 s (patients)	3. Baseline correction; averaging
		4. 15 s before/after walking	4. OxyHb
			5. Acceleration phase = 6 s after starting treadmill; steady phase = 6 s during steady speed
Mihara et al.^94^	– Healthy young adults (n=15; 29.4±6.7)	1. Quiet standing	1. N/A (arbitrary unit)
	• Warned before perturbations vs. baseline; unwarned before perturbations vs. baseline; warned vs. unwarned	2. Time before next perturbation (ERD)	2. HPF at 0.05 Hz
		3. 20 to 30×; 1 s	3. Gaussian HRF; averaging
		4. 5 to 20 s between trials (randomized) / 4 to 5 min after block	4. OxyHb
			5. Around perturbation
Mihara et al.^95^	– Stroke patients (n=20; 61.6±11.9)	1. Quiet standing	1. N/A (arbitrary unit)
	• Balance perturbations	2. Time before next perturbation (ERD)	2. HPF at 0.03 Hz
		3. 15×; 1 s	3. Two-parameter gamma HRF
		4. 5 to 15 s between trials (randomized)	4. OxyHb
			5. Around perturbations
Mirelman et al.^77^	– Young, healthy adults (n=23; 30.9±3.7)	1. Quiet standing	1. Age-dependent value ($4.99+0.067\times {\text{age}}^{0.814}$)
	• Standing vs. DTS vs. NW vs. DTW	2. 20 s before walking	2. LPF at 0.14 Hz; continuous wavelet transform
		3. 5×; 30 m	3. Baseline correction; averaging
		4. 20 s before/after trial	4. OxyHb
			5. Entire task phase
Miyai et al.^107^	– Healthy young adults (n=8; 35±8, 24 to 56)	1. Quiet standing	1. N/A (arbitrary unit)
	• NW vs. standing	2. 30 s	2. HPF at 0.03 Hz
		3. 5×; 30 s	3. Linear interpolation; averaging
		4. 30 s between trials	4. Oxy- and deoxyHb
			5. Entire task phase
Miyai et al.^61^	– Stroke patients (n=6; 57±13)	1. Quiet standing	1. N/A (arbitrary unit)
	• Walking with mechanical assistance vs. walking with facilitation technique	2. Middle 20 s	2. HPF at 0.03 Hz
		3. 4×; 30 s	3. Linear interpolation; baseline correction; averaging
		4. 30 s between trials	4. OxyHb
			5. Last 20 s of task phase
Miyai et al.^60^	– Stroke patients (n=8; 57±12)	1. Quiet standing	1. N/A (arbitrary unit)
	• Before/after 2 months rehabilitation	2. Middle 20 s	2. HPF at 0.03 Hz
		3. 4×; 30 s	3. Linear interpolation; baseline correction; averaging
		4. 30 s between trials	4. OxyHb
			5. Last 20 s of task phase
Miyai et al.^59^	– Stroke patients with hemiparesis (n=6; 57±6)	1. Quiet standing	1. N/A (arbitrary unit)
	– Healthy controls (n=6, 53±11)	2. Middle 20 s	2. HPF at 0.03 Hz
	• Walking with weight support vs. walking without weight support	3. 4×; 30 s	3. Linear interpolation; baseline correction; averaging
		4. 30 s between trials	4. OxyHb
			5. Last 20 s of task phase
Nieuwhof et al.^81^	– Parkinson patients (n=14; 71.2±5.4)	1. Quiet standing	1. Constant value (6.0)
	• DTW (with different tasks)	2. Last 5 s of standing	2. LPF at 0.1 Hz; visual inspected
		3. 5×; 40 s	3. Baseline correction; averaging
		4. 20 s between trials / 1 to 2 min between blocks	4. OxyHb and deoxyHb
			5. Entire task phase
Osofundiya et al.^87^	– Obese old adults (n=10; 80.6±6.79)	1. Quiet standing	1. Constant value (6.0)
	– nonobese old adults (n=10; 80.6±7.50)	2. 10 s	2. Not reported
	• Quiet sitting vs. NW vs. precision walking vs. DTW	3. 8× a 30 s	3. Baseline correction; averaging
		4. 10 s between trials	4. OxyHb and HbT
			5. Entire task phase
Saitou et al.^78^	– Hemiplegic stroke patients (n=44; 66±9.3)	1. Quiet standing	1. Constant value (5.9)
	• Different tasks (e.g., calculation, pulley, we only consider walking vs. baseline)	2. 5 min	2. Not reported
		3. 1×; 5 min	3. Averaging
		4. 5 min	4. OxyHb; CBV; COV
			5. Entire task phase
Suzuki et al.^62^	– Healthy, young adults (n=9; 28.1±7.4, 22 to 46)	1. Quiet standing	1. N/A (arbitrary unit)
	• Walking at different speeds	2. First 13 s	2. HPF at 0.03 Hz
		3. 3×; 90 s	3. Linear interpolation; baseline correction; averaging
		4. 30 s between trials	4. Oxy- and deoxyHb; regional cortical activation ratio (oxy Hb of the specific channel divided by oxyHb of all 42 channels multiplied by 100)
			5. 13.5 s in task phase
Suzuki et al.^67^	– Healthy, young adults (n=7; 31.3±4.8, 24 to 45)	1. Quiet standing	1. Not relevant (arbitrary unit)
	• Walking with vs. without verbal preadvice	2. 10 s before walking	2. HPF at 0.03 Hz
		3. 4×; 40 s	3. Baseline normalization; averaging
		4. 10 to 25 s between trials (randomized order)	4. Oxy- and deoxyHb
			5. First 10 s of task phase
Takeuchi et al.^89^	– Young healthy adults (n=16; 25.9±4.4, 20 to 33)	1. Walking	1. Constant value (no details reported)
	– Healthy older adults (n=15; 71.7±3.3, 65 to 78)	2. 30 s	2. Spike rejection (artifact with more than 3 SD); 5 s moving average; bandpass filter (LPF at 0.5 Hz; HPF at 0.01 Hz)
	• Walking vs. walking with smartphone	3. 15×; 10 s	3. Baseline normalization; averaging
		4. No rest	4. OxyHb
			5. Entire task phase
Takakura et al.^96^	– Healthy young adults (n=11; 33.4±7.4)	1. Quiet standing	1. Constant value (1.0)
	• SOT conditions	2. 20 s before task	2. Bandpass Fourier filter (0.01 to 0.1 Hz)
		3. 3×; 20 s	3. Averaging
		4. Few minutes after 3 trials	4. OxyHb
			5. Entire task phase
Verghese et al.^83^	– Older adults (n=166; 75±6.1)	1. Quiet standing	1. Constant value (6.0)
	– NW vs. DTW vs. standing	2. 10 s (counting silently forward in steps of 1)	2. LPF at 0.14 Hz; noisy channels excluded (dark current condition or saturation); visual inspected
		3. 3× walking loops (= 6× straight walks a 14 ft.) / standing for 30 s)	3. Baseline normalization; averaging
		4. “Short break” reported	4. OxyHb
			5. Entire task phase
Wang et al.^104^	– Healthy young adults (n=22; 24.4±1.6)	1. Sitting (eyes closed)	1. Age-dependent constant value (WL: 760 nm=5.91; WL: 850=5.40)
	– Healthy older adults (n=39; 70.5±7.7)	2. 20 min	2. Bandpass filter (0.005 to 2 HZ)
	• Standing connectivity differences healthy young and healthy old adults	3. 1×; 10 min	3. Wavelet phase coherence analysis
		4. No rest	4. OxyHb
			5. Entire task time

Abbreviations: deoxyHb, deoxygenated hemoglobin; DTS, dual-task standing; DTW, dual-task walking; ft., feet; HC, healthy controls; HPF, high-pass filter; HRF, hemodynamic response function; LPF, low-pass filter; MDL, minimum description length; MRI, magnetic resonance imaging; N/A, not applicable; NW, normal walking; NGA, neurological gait abnormalities; oxyHb, oxygenated hemoglobin; PCA, principal component analysis; RAW, robot assisted walking; SOT, sensory organization test; ST, stepping; TOI, tissue oxygenation index; and vs., versus.

#### 3.1.2 Overground walking

Twenty-one studies conducting overground walking, quantified baseline brain activation in a standing position.^66^^,^^69^^–^^88^ In contrast, two studies assessed baseline brain activation while walking^89^ or during a predefined time period prior to a freezing of gait event (FOG; a sudden, brief inability to start movement or to continue rhythmic, repeated movements despite the internal intention to move).^90^ The duration to assess baseline brain activity ranged between 5 s^69^^,^^73^^,^^76^^,^^81^ and 5 min.^78^ Most studies used 10 s to quantify baseline brain activity.^66^^,^^70^^–^^72^^,^^74^^,^^75^^,^^82^^–^^88^ Interestingly, Holtzer et al.^74^^,^^75^^,^^83^^–^^85^ asked their participants to conduct a simple counting task (in steps of 1) during the baseline condition (for an overview see Table 1).

#### 3.1.3 Postural tasks

In postural research, 13 studies assessed baseline brain activity during quiet standing.^91^^–^^103^ The temporal duration to quantify baseline brain activity ranged from 2^93^ to 60 s.^97^ In most studies, data of 30 s^98^^–^^100^ or a few seconds before starting the next trial^91^^,^^94^^,^^95^^,^^102^ were used to assess baseline brain activation. In addition, Wang et al.^104^ used 20 min quiet sitting to measure baseline connectivity (for an overview see Table 1).

### 3.2 Number and Duration of Trials and Rest Phases

#### 3.2.1 Treadmill walking

The studies that used a treadmill for the walking condition^53^^–^^68^^,^^105^^–^^107^ are shown in Table 1. Per task, a minimum of 2 trials^105^ and a maximum of 10 trials^54^^–^^56^ were performed. Most studies used three to five trials to assess task-relevant cortical activity.^53^^,^^57^^,^^59^^–^^62^^,^^64^^,^^64^^,^^65^^,^^67^^,^^106^^,^^107^ Task phases were set to 30 s in the majority of the studies,^55^^–^^61^^,^^65^^,^^68^^,^^105^^–^^107^ but Harada et al.,^53^ Kim et al.,^106^ and Mihara et al.^58^ used 60 s, Koenraadt et al.^54^ used 35 s, Metzger et al.^64^ used 45 s, Suzuki et al.^67^ used 40 s, Suzuki et al.^62^ used 90 s, and Fraser et al.^63^ used 120 s. The time of the rest phases ranged in most studies between 25 and 60 s.^53^^–^^57^^,^^59^^–^^62^^,^^65^^,^^68^^,^^106^^,^^107^ Additionally, rest times of 15 s prior to^58^^,^^64^ and after each walking trial^58^ were reported while Suzuki et al.^67^ implemented 10 to 25 s between trials (for an overview see Table 1).

#### 3.2.2 Overground walking

Twenty-three studies investigated cortical hemodynamic responses during overground walking.^66^^,^^69^^–^^90^ For each condition, 1,^70^^,^^79^ 3,^76^ 4,^87^ 5,^66^^,^^69^^,^^71^^,^^77^^,^^80^^,^^81^^,^^86^ 6,^73^^–^^75^^,^^82^^–^^85^ and 15 walks were used.^89^ Either the time for each task phase ranged between 10^69^ and 120 s^66^ or the participants were asked to walk a predetermined distance ranging between ∼4^73^^–^^75^^,^^82^^,^^83^^,^^85^ and 90 m.^86^ The resting phases prior to and after each trial lasted 20 s^77^^,^^79^ or 60 s^76^ and 10 s^87^ or 30 s between the trials.^72^ Two studies used 20 s of rest between successive trials and 1 to 2 min of rest between successive task blocks.^80^^,^^81^ Furthermore, in three studies, a rest of 2 min was used^66^^,^^86^^,^^90^ while one study allowed participants to rest 5^88^ or 30 min between tasks^71^ (for an overview see Table 1).

#### 3.2.3 Postural tasks

Regarding the examination of brain activity during a sensory organization test (SOT; a balance test using quantitatively different visual, proprioceptive, and vestibular cues to assess the quality of postural stance stability), two trials,^92^ three trials,^96^ or four trials were conducted^103^ which lasted 45,^92^ 40,^103^ or 20 s.^96^ The participants of the three studies using mechanical perturbations performed 15^95^^,^^102^ to 30 trials^94^ with a randomized perturbation duration of 5 to 20 s.^94^^,^^95^^,^^102^ In semivirtual reality, seven trials with a task phase duration of 45 s were used.^98^ The rest between task phases depended on the conducted tasks (see Table 1) and ranged between 4 and 20 s.^91^^,^^94^^,^^95^^,^^101^ In other studies, a rest of 1^103^ or 2 min was included.^92^^,^^98^^,^^99^ To avoid fatigue, resting times after some trials that lasted a few minutes were common^91^^,^^92^^,^^94^ (for an overview see Table 1).

### 3.3 Source–Detector Separation

**Fig. 2:**
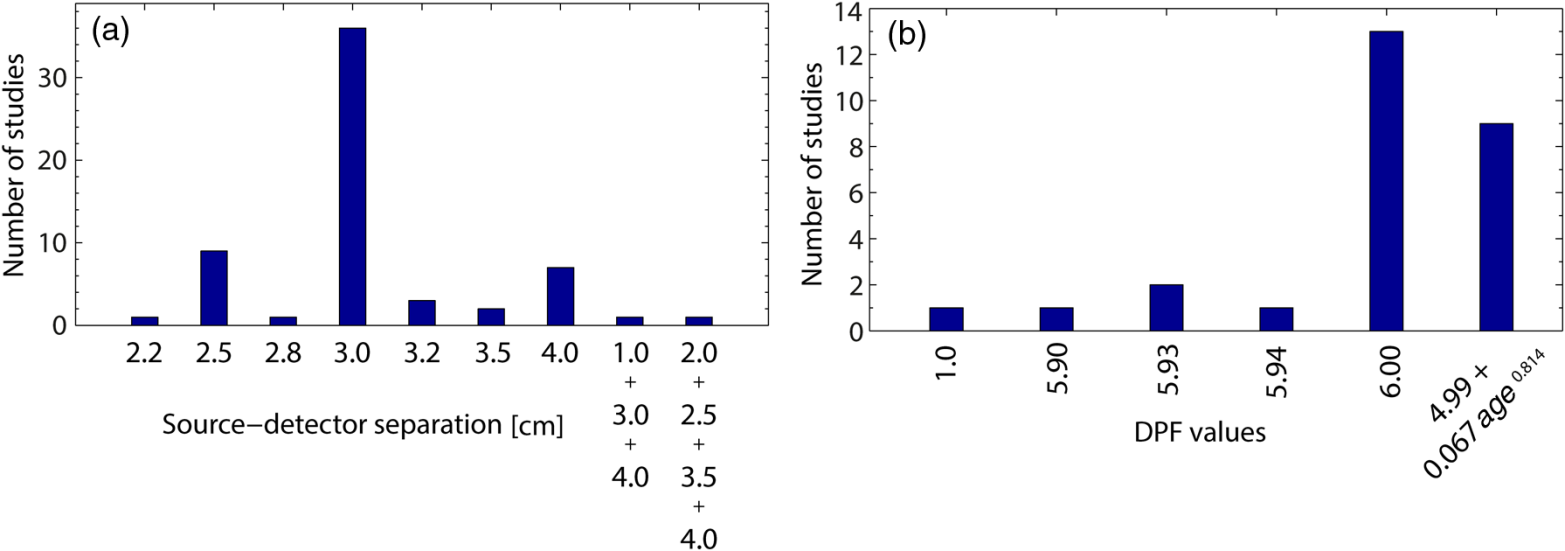
Overview on (a) used source–detector separation and (b) DPF values in the reviewed studies.

The closest distances between the optodes (source and detector) were reported to be ∼1  cm, which was used as a short separation channel^54^ and was followed by an interoptode distance of 2.5 cm.^73^^–^^75^^,^^82^^–^^85^^,^^93^^,^^105^ Three studies used 3.2 cm,^91^^,^^92^^,^^97^ and two studies used 3.5 cm.^80^^,^^81^^,^^90^ Another seven studies used 4 cm.^70^^,^^71^^,^^78^^–^^81^^,^^104^ One study used a different distance between source and detector (1, 3, and 4 cm)^54^ and another one used multidistance measurement (2.0, 2.5, 3.5, and 4.0 cm).^68^ The remaining 36 studies set the interoptode distances at 3 cm.^53^^,^^55^^–^^62^^,^^64^^–^^67^^,^^69^^,^^72^^,^^76^^,^^77^^,^^80^^,^^81^^,^^86^^–^^89^^,^^94^^–^^96^^,^^98^^–^^104^^,^^106^^–^^108^ An overview on used source–detector is shown in Fig. 2(a).

### 3.4 Placement of the Optodes

The majority of studies used the international “10-20 EEG system” for the placement of the optodes.^53^^–^^57^^,^^59^^–^^65^^,^^67^^–^^77^^,^^79^^,^^81^^–^^83^^,^^85^^,^^87^^–^^108^ In some studies, an additional three-dimensional (3-D)-digitizer was applied^69^^,^^76^^,^^94^^–^^96^^,^^106^^,^^108^ or an MRI scan was conducted^59^^–^^62^^,^^67^^,^^90^^,^^94^^–^^96^^,^^102^^,^^107^ to coregister optode positions on the head. Other placement strategies (placing optodes on the forehead) were applied in four studies.^58^^,^^66^^,^^80^^,^^86^

### 3.5 Differential Path Length Factor

The differential path length factor (DPF) is a scaling factor that specifies how many times the detected light has traveled farther than the source–detector separation through the brain.^109^^,^^110^ In 21 studies, constant DPF values were used^63^^,^^69^^–^^71^^,^^73^^–^^76^^,^^78^^,^^81^^–^^85^^,^^87^^–^^89^^,^^93^^,^^96^^,^^105^^,^^108^ whereas nine studies used age-dependent DPF values.^57^^,^^65^^,^^77^^,^^79^^,^^80^^,^^90^^,^^98^^,^^99^^,^^104^

For constant DPF, values of 1.0,^96^ 5.9,^78^ 5.93,^70^^,^^71^ 5.94,^88^ and 6.0^73^^–^^76^^,^^81^^–^^85^^,^^87^^,^^93^^,^^105^^,^^108^ were used, while age-dependent DPF values were calculated according to the formula ($\mathrm{DPF}=4.99+0.067\times {\text{age}}^{0.814}$).^57^^,^^65^^,^^77^^,^^79^^,^^80^^,^^90^^,^^98^^,^^99^ An overview on used DPF values is provided in Fig. 2(b). In other studies, arbitrary units,^53^^–^^56^^,^^58^^–^^62^^,^^64^^,^^67^^,^^72^^,^^94^^,^^95^^,^^100^^–^^102^^,^^107^ tissue oxygenation index [TOI; the ratio of oxyHb to total hemoglobin (sum of oxy- and deoxyHb)],^66^^,^^86^ image reconstruction,^91^^,^^92^^,^^97^^,^^103^ or absolute values^68^ were used, which are not dependent on specific DPF values.

### 3.6 Data Processing: Signal Filtering and Movement Artifact Removal

Twenty-one studies applied a low-pass filter (LPF) to their data,^54^^,^^57^^,^^65^^,^^71^^–^^75^^,^^77^^,^^79^^,^^81^^–^^83^^,^^85^^,^^88^^,^^90^^,^^93^^,^^98^^–^^101^ 14 studies used a high-pass filter (HPF)^53^^,^^55^^,^^56^^,^^59^^–^^62^^,^^67^^,^^94^^,^^95^^,^^100^^–^^102^^,^^107^ and 5 studies used a bandpass filter.^76^^,^^80^^,^^89^^,^^96^^,^^104^ Most studies applied an LPF with a cut-off frequency around 0.1 Hz^71^^,^^73^^–^^75^^,^^77^^,^^80^^–^^85^^,^^88^^,^^90^^,^^93^^,^^98^^,^^99^^,^^108^ whereas some studies used an LPF with a cut-off frequency at 0.05,^72^ 0.67,^57^^,^^65^ 0.5,^100^^,^^101^ and 1 Hz^54^ (for an overview see Table 1). Eight studies applied an HPF with a cut-off frequency at 0.03 Hz,^53^^,^^59^^–^^62^^,^^67^^,^^95^^,^^107^ six studies at 0.01 Hz,^54^^–^^56^^,^^80^^,^^100^^,^^101^ and one study at 0.05^94^ or 0.001 Hz.^108^ Furthermore, eight studies used the moving average method^55^^–^^57^^,^^64^^,^^68^^,^^89^^,^^100^^,^^101^ to smooth their data. Filter methods based on principal component analysis (PCA) were conducted in six studies^55^^,^^56^^,^^73^^,^^76^^,^^100^^,^^102^ and a spike artifact correction was used in three studies.^68^^,^^76^^,^^89^ Few studies applied HRF filter,^105^ an autoregressive model with prewhitened iterative reweighted least square algorithms,^103^ wavelet filter,^80^^,^^105^^,^^106^ Gaussian smoothing,^106^ and correlation-based signal improvement (CBSI).^64^^,^^80^ A visual inspection of data was reported in 13 studies.^68^^,^^74^^,^^75^^,^^81^^–^^85^^,^^88^^,^^93^^,^^97^^,^^108^^,^^111^

### 3.7 Data Processing: Correction for Physiological Artifacts

One study applied short separation channels^54^ and one study used multidistance measurements^68^ to correct for superficial blood flow. For multidistance measurements or short separation channels, normally lower source–detector separation (<1.5  cm) is chosen, which is used to probe extracerebral noise. Furthermore, the following additional physiological parameters were measured to take into account systemic physiological artifacts: (1) heart rate,^53^^,^^57^^,^^59^^–^^62^^,^^71^^,^^87^^,^^98^^,^^99^^,^^107^ (2) blood pressure,^53^^,^^54^^,^^57^^,^^59^^–^^62^^,^^71^^,^^107^ and (3) arterial oxygen saturation.^59^^–^^62^^,^^107^ The usage of filter methods based on PCA, which can be useful for the correction of motion and physiological noise, was used in six studies.^55^^,^^56^^,^^73^^,^^76^^,^^100^^,^^102^

### 3.8 Data Processing: Final Data Processing and Statistical Analysis

Twenty-three of the reviewed studies used a baseline correction^53^^,^^55^^,^^56^^,^^58^^–^^62^^,^^64^^,^^65^^,^^69^^–^^71^^,^^77^^,^^79^^–^^81^^,^^87^^,^^90^^,^^96^^,^^100^^,^^101^^,^^105^ and 14 studies conducted a baseline normalization.^53^^,^^54^^,^^67^^,^^73^^–^^75^^,^^82^^–^^85^^,^^89^^,^^93^^,^^101^^,^^108^ Furthermore, almost all studies computed an average of (1) all trials and (2) across the channels of a specific ROI.^53^^–^^65^^,^^68^^–^^77^^,^^80^^–^^85^^,^^87^^,^^89^^–^^91^^,^^93^^–^^96^^,^^98^^–^^101^^,^^103^^,^^107^

In addition, linear interpolations were used in the studies of Miyai et al. and Suzuki et al.^59^^–^^62^^,^^107^ A method based on moving standard deviation and spline interpolation was applied by Beurskens et al.^105^ Three studies applied discrete cosine transform terms.^91^^,^^92^^,^^97^

Canonical hemodynamic response function was conducted in two studies^105^^,^^106^ that examined cortical activation during walking. Studies researching postural tasks used either a gamma hemodynamic response function^91^^,^^92^^,^^95^^,^^102^^,^^103^ or a Gaussian hemodynamic response function.^94^ A wavelet coherence analysis was used in one study.^104^

Five studies divided their task phase in different time periods,^58^^,^^68^^,^^76^^,^^86^^,^^90^ 18 studies used predetermined time intervals inside the task phase,^53^^,^^54^^,^^57^^,^^59^^–^^62^^,^^65^^,^^67^^,^^69^^–^^71^^,^^73^^,^^93^^,^^94^^,^^98^^–^^100^ and 24 studies used the entire task phase for analysis.^55^^,^^56^^,^^63^^,^^64^^,^^72^^,^^74^^,^^75^^,^^77^^,^^80^^,^^82^^,^^83^^,^^85^^,^^87^^,^^89^^,^^91^^,^^92^^,^^96^^,^^97^^,^^101^^–^^103^^,^^106^^–^^108^

The statistical analysis was performed in 47 studies with parametric^53^^,^^54^^,^^56^^–^^70^^,^^73^^–^^77^^,^^79^^,^^80^^,^^82^^–^^89^^,^^91^^–^^99^^,^^103^^–^^108^ and in one study with nonparametric methods.^81^ Eight studies used parametric and nonparametric methods^55^^,^^71^^,^^72^^,^^78^^,^^90^^,^^100^^–^^102^ (see Table 1).

### 3.9 Markers for the Assessment of Cortical Activation

The majority of reviewed studies used changes of oxyHb to assess brain activation.^53^^,^^57^^–^^62^^,^^68^^,^^70^^–^^75^^,^^77^^–^^80^^,^^82^^–^^85^^,^^89^^,^^90^^,^^93^^–^^96^^,^^101^^,^^104^^,^^106^ Furthermore, 21 studies used both oxyHb and deoxyHb to quantify the activation of the region of interest.^54^^–^^56^^,^^63^^–^^65^^,^^67^^,^^69^^,^^81^^,^^88^^,^^91^^,^^92^^,^^97^^–^^100^^,^^102^^,^^103^^,^^105^^,^^107^^,^^108^ Only Clark et al.^66^^,^^86^ used the TOI, which is the ratio of oxygenated to total tissue hemoglobin, to evaluate cortical activation. In addition, Lu et al.^76^ used Hb diff (oxyHb – deoxyHb) for the quantification of cortical activation. Furthermore, one study used a cortical activation ratio^62^ to measure brain activation (for an overview see Table 1).

## 4 Results: Main Findings of the Studies

In the following sections, we will provide an overview about the main findings of the reviewed studies. The results section is divided into outcomes of walking and postural tasks.

### 4.1 Walking

Walking was associated with a higher activation of prefrontal cortex (PFC),^53^^,^^54^^,^^57^^,^^58^^,^^67^ presupplementary motor area,^53^^,^^67^ premotor cortex (PMC),^53^^,^^106^ supplementary motor area (SMA),^53^^–^^55^^,^^58^^,^^67^^,^^106^^,^^107^ and sensorimotor cortex (SMC).^53^^,^^58^^,^^67^^,^^106^ A higher PFC activation was observed in persons with low gait capacity,^53^ high perceived stress,^84^ high perceived fatigue,^85^ high risk of falling,^83^ ataxic gait,^70^^,^^71^ and patients with Parkinson’s disease^80^^,^^90^ during walking. Moreover, higher activation of precentral gyrus (PrG), postcentral gyrus (PoG), and superior parietal lobule (SPL) was observed in children with cerebral palsy^56^ and in stroke patients in the nonaffected hemisphere in the PFC,^58^ SMA,^58^^,^^61^ and SMC.^61^ During dual-task walking (e.g., walking and solving an additional cognitive or motor task), the PFC exhibited an enhanced activation in stroke patients,^65^ patients with multiple sclerosis,^82^ patients with Parkinson’s disease,^81^ obese adults,^87^ older adults with mild cognitive impairments,^72^ old adults with mobility deficits,^66^^,^^86^ and healthy older^63^^,^^65^^,^^73^^–^^75^^,^^82^ and young adults.^57^^,^^63^^,^^64^^,^^73^^,^^76^^,^^77^ In comparison to young adults, older adults exhibited a higher^73^ or similar^63^ PFC activation during dual-task walking. The activation of PFC during dual-task walking was associated with the performance in motor tasks,^75^^,^^77^^,^^89^^,^^105^ cognitive tasks,^75^^,^^77^^,^^89^ and neuropsychological tests.^72^ In single task walking, PFC activation positively correlated with the neuropsychological performance in healthy older persons^68^ and with motor performance in neurologically diseased persons.^70^^,^^71^ A decrease in PFC activation was observed in younger adults while walking and solving a working memory task^79^^,^^88^ and in healthy seniors while solving a complex visual task.^105^ Interestingly, the activation of PFC in older adults is decreased after a motor intervention^68^ and when textured insoles were used or barefoot walking was conducted.^66^ In contrast, the inpatient intervention in stroke patients enhanced PMC activation during walking.^60^ Additionally, an increase of motor complexity due to the increase in walking speed led to a pronounced activation of PFC,^62^ SMA,^53^ and Broca area,^64^ whereas a decrease of motor complexity due to body weight support induced a decrease in SMC activation.^59^

### 4.2 Postural Tasks

In balance tasks, the activation of PFC,^91^^,^^98^^,^^99^ SMA,^101^^,^^102^ and superior temporal gyrus^97^ was modulated by task difficulty and by age-related processes.^104^ Furthermore, an increased PFC activation was observed during standing in young adults with postconcussion symptoms,^108^ in patients with Parkinson’s disease^93^ or in stroke patients’ in the affected^95^^,^^102^ and unaffected hemisphere.^95^ Furthermore, stroke patients showed a stronger activation in PMC and parietal areas concerning the unaffected hemisphere.^95^ After the rehabilitation program, the same patients showed a decreased activation of PMC and parietal areas but a bilateral increase in PFC and SMA activations.^102^

During the SOT, different sensory information changes the functional connectivity of brain areas^96^^,^^103^ and induced activation changes especially in superior marginal gyrus,^92^^,^^96^ operculum,^96^ temporal–parietal areas,^103^ and occipital regions.^103^ Additionally, correlation between balance performance and the activation of PFC^95^^,^^102^ and SMA was observed.^95^^,^^100^^,^^102^

## 5 Discussion

fNIRS is a relatively new neuroimaging technique that has attracted attention in scientists who examine neuromotor control. This resulted in a considerable magnitude of published studies. However, a summarization and evaluation that can help to improve future experimental protocols was still required. In the first part of the discussion section, we will discuss the findings about study designs, fNIRS configurations and data processing steps to come closer to more standardized protocols that are not available at this moment.^27^^,^^112^ In the second part, the main findings of the reviewed studies are discussed.

### 5.1 Discussion: Methodology

#### 5.1.1 Baseline condition and duration

The majority of studies with walking or postural tasks assessed baseline brain activation in quiet standing. Interestingly, Holtzer et al.^74^^,^^75^ used a silent counting task to avoid mind wandering. Mind wandering occurs up to 50% of the waking hours^113^ for instance during driving^114^^,^^115^ especially when perceptual requirements are low.^116^ Moreover, the wandering of the mind is characterized by the processing of task unrelated thoughts such as worrying about the past or future,^117^ which evokes a stronger activation of default networks^118^ and hence changes the activation in PFC areas.^119^^,^^120^ In addition, it was shown by Durantin et al.^120^ that fNIRS is sensitive to detect mind wandering. Based on these assumptions, it is possible that mind wandering influences the cortical activation during baseline (and maybe motor control) affecting further analyzation processes. Hence, it might be advantageous to use the approach of Holtzer et al.,^74^^,^^75^ which eventually minimizes the detrimental effect of mind wandering on cortical activation and leads to a more standardized baseline assessment. However, before the usage of this simple counting task can be recommended, further research should investigate its influence on cortical activation patterns including examination of enhanced reproducibility.

#### 5.1.2 Number and duration of trials and rest phases

Our results revealed that the number of trials and their durations varied across the studies evaluating walking or postural tasks. The most common time interval was set to 30 s. However, we are unaware of a study investigating the influence of measurement strategy (e.g., required number of trials to achieve a sufficient reproducibility). Hence, further methodological investigations to optimize fNIRS measurement protocols are needed. Moreover, the duration and number of the trials depend on the aim of the study. Longer measurement durations may be useful to study the contribution of different areas in the temporal course of movement execution. In contrast, longer measurement durations could result in motor fatigue. Motor fatigue does diminish performance for example in postural tasks^121^^–^^126^ and would hence change underlying motor control processes. This again could potentially evoke altered hemodynamic responses, which were observed after cognitive fatigue.^127^ However, research examining the interplay between a specific gross motor task and hemodynamic responses as a function of physical fatigue level has not been conducted yet.

Another interesting point influencing the trial duration is the combination of analysis methods. From a movement scientific view, the analysis of gait features (especially gait variability and stability) gives an insight in the central organization of motor control processes^128^^–^^131^ and those are useful to detect risk groups such as fallers.^132^^,^^133^ To reliably assess gait variability or stability, a larger number of strides is required^134^^,^^135^ and as a consequence, a sufficiently long time period (in which an adequate number of strides can be undertaken) of the trial duration has to be recorded. The rest phase durations in included studies have varying temporal ranges. In general, empirical evidence suggests that refraction time or time with reduced responsiveness lasted for almost the same duration as stimulus time.^136^ Hence, we recommend to include intertrial rest intervals with at least the same duration as the task period, especially in block design studies.

#### 5.1.3 Source–detector separation

The separation of source to detector is one important aspect for penetration depth^27^^,^^34^ and the influence of extracerebral signals.^34^^,^^137^ Our results indicated that 3 cm was the most commonly used distance in the reviewed studies. In the literature, different recommendations about optimal source–detector separation exist. While some authors recommend 4 cm,^34^ other collectives recommend 3 cm.^138^^,^^139^ In addition, especially in children or infants shorter interoptode distance (>2.0  cm) is recommended for usage.^22^^,^^139^ The issue of the optimal separation between source and detector is a controversial debate because different third variables such as different colors of the participant’s skin and/or hair used wavelengths and head size could influence penetration depth.^34^^,^^140^ Furthermore, the varying thickness of scalps, skulls, and cerebrospinal fluids in individuals and cortical regions^141^^–^^143^ could influence the penetration depth and the sensitivity to hemodynamic changes in cortical layer.^142^^–^^144^

Remarkably, a longer source–detector separation leads to a greater contribution of cerebral than extracerebral layer to obtain hemodynamics signals.^145^^–^^148^ The penetration depth of light is less than half of the interoptode distance^147^ causing short channel distances to cover only signals from noncerebral compartments.^137^^,^^141^ For instance, at the source–detector separation of 3 cm, the contribution of the gray matter to the light absorption is estimated to range from about 20% to 30%.^149^ Moreover, Kohri et al.^150^ observed that at source–detector separation of 2, 3, and 4 cm, the cerebral tissue contributes to 33%, 55%, and 69% to the optical signal. Hence, we recommend that the source–detector separation should be greater than 3 cm to enhance the contribution of cerebral cortical layer to the optical signal.

#### 5.1.4 Placement of optodes

The majority of the studies used the 10 to 20 EEG systems to place the optodes. This standardized location system ensures the comparability among the different studies. The additionally used 3-D digitizer or individual MRI scan improves the registration of channels to specific brain areas. Based on the data we recommend for optode placement the usage of the 10 to 20 EEG systems to ensure the comparability among studies.

#### 5.1.5 Differential path length factor

Our results show that most studies used constant DPF with a value of 6. The usage of a constant DPF value seems not always appropriate because the brain undergoes age-related changes of gray and white matter,^151^^,^^152^ intracranial volume,^153^ and cerebral volume as well as blood flow^154^, which may affect DPF.^155^ Furthermore, methodological studies show that DPF values are (1) age-dependent and subject-specific,^110^^,^^155^^,^^156^ (2) wavelength-dependent,^110^^,^^155^^,^^157^ and (3) cortex region-dependent.^110^^,^^155^^,^^158^^–^^160^ Hence, it seems favorable to calculate specific DPF values to enhance the measurement accuracy in age-groups in which formulas to calculate age-specific DPF values are available (adults under 50 years).^110^^,^^155^ Otherwise, “arbitrary units,”^161^ TOI,^162^^–^^164^ or absolute values^137^^,^^163^^,^^165^ could be used since those do not depend on a specific DPF value. In addition, it is suggested that the calculation of effect sizes is useful to deal with the DPF issue.^166^ However, additional research is strongly needed that provides a formula to calculate DPF values for specific age-groups (adults older than 50 years) dependent on wavelength and cortex region. In our opinion, the optimal approach to quantify DPF, taking the dependency of DPF regarding subject, age, wavelength, and cortex region into account, is the direct quantification of DPF using frequency or time-domain NIRS.

#### 5.1.6 Data processing: signal filtering and movement artifact removal

In sum, either LPFs or HPFs were commonly applied in the reviewed studies to remove noise and drifts. Most of the studies used a cut-off frequency for LPF around 0.1 Hz and HPF around 0.01 Hz. The reviews of Brigadoi et al.,^167^ Cooper et al.,^168^ and Gervain et al.^40^ recommended to use a bandpass filter (consisting of both LPFs and HPFs) with cut-off frequencies at 0.5 (LPF) and 0.01 Hz (HPF). The bandpass filtering should be used carefully to avoid accidental removal of stimulus-dependent hemodynamic response signals.^111^ Hence, a higher cut-off frequency at 0.5 Hz (LPF) in conjunction with other more sophisticated filter methods is recommended to be used for the removal of movement and physiological noise.^111^^,^^167^^,^^168^ Different methods such as PCA,^169^^–^^171^ task-related component analysis,^172^^–^^174^ CBSI,^175^ wavelet-based filters,^171^^,^^176^^–^^179^ autoregressive algorithm-based filters,^180^ Kalman filter,^181^ and Wiener filter^182^ are proposed for the filtering of fNIRS data. Interestingly, Nozawa et al.^183^ suggested that effectiveness of motion correction filter methods depends on subject and task. However, reviews comparing a variety of filter methods recommend the additional application of wavelet filter^167^^,^^168^ or spline technique.^168^ These filter methods were occasionally applied in reviewed studies^80^^,^^105^^,^^106^ leaving potential to optimize the filtering processes in further studies. Based on these assumptions, we recommend the usage of a bandpass filter and wavelet filter to reduce motion artifacts. If there are sudden shifts in the data (baseline shift), the approach developed by Scholkmann et al.^184^ can be useful to remove them.

#### 5.1.7 Data processing: correction for physiological artifacts

Twelve studies recorded physiological signals such as heart rate, blood pressure, or arterial oxygenation saturation parallel to the fNIRS signals. Task-related systematic changes in heart rate, respiration rate, or blood pressure are known to influence the fNIRS signal and may cause false-positive results.^45^ For instance, often unconsidered factors such as adding of speech as a task (e.g., in dual-task paradigms) lead to changes in partial pressure of end-tidal carbon dioxide, which influences cerebral hemodynamics and masked neuronal-induced activity changes.^185^^,^^186^ Hence, to improve the accuracy of fNIRS, the recording and elimination of systemic physiological changes seems necessary.^45^^,^^187^^,^^188^ The signals of additional physiological measures could be useful for filtering of fNIRS signal^189^^,^^190^ or to ensure the absence of systematic physiological differences among the experimental conditions.^87^ In addition, some measures such as heart rate variability could be used to study the interplay between the central (fNIRS) and the autonomic (e.g., heart rate variability) nervous system.^120^^,^^191^ Furthermore, filter methods based on PCA and independent component analysis, which were applied in six studies,^55^^,^^56^^,^^73^^,^^76^^,^^100^^,^^102^ could be used to remove movement-related^167^ or physiological artifacts.^169^^,^^170^^,^^192^^–^^195^ In addition to the other filter methods,^196^^,^^197^ a more “direct” approach to reduce extracerebral noise is the use of short separation channels or multidistance technique^198^^–^^200^, which were applied in only two of the reviewed studies.^54^^,^^68^ Short separation channels have a small distance between source and detector to record extra cerebral signals, such as superficial blood flow.^141^^,^^198^^,^^201^ These extracerebral signals are used to filter the remaining fNIRS data. Previous studies revealed that the application of short separation channels is powerful in reducing extracerebral noise^141^^,^^145^^,^^200^^,^^202^^–^^208^, which contaminates fNIRS signals.^45^^,^^199^^,^^201^^,^^209^^–^^214^ The optimal distance between short separation channels varied across different cortex regions^141^^,^^202^ but should be generally <1  cm for measurement on the head of adult humans. Hence, further development and implementation of short separation channels (multidistance technique) could enhance the accuracy of fNIRS measurements and have to be considered whenever technically possible.

#### 5.1.8 Data processing: final data processing and statistical analysis

Most studies used baseline normalization and baseline correction to circumvent the influence of different path lengths factors.^166^ Furthermore, averaging of channels across trials and in specified ROIs was common practice in the reviewed studies.

Some studies divided their task phase in different time periods, which seems useful for studying the contribution of cortical areas in different temporal periods during task execution. Therefore, attention should be paid to the temporal delay of ∼2 to 5 s in hemodynamic response.^69^^,^^107^^,^^139^

The majority of the reviewed studies used simple statistics based on processing mean values over the task period. This approach, however, tends to result in a loss of acquisition of information because it does not consider the temporal shape of the fNIRS signal.^192^ Hence, some authors suggest that the analysis of fNIRS data with general linear models is more favorable.^192^^,^^215^ However, the choice of the statistical analysis methods should depend on the research question and the experimental design.^216^ For instance, in an event-related design, the application of a general linear model is a valid technique^216^ whereas simple statistics might also be appropriate (and commonly used^192^) especially in studies utilizing block designs.^55^^,^^56^^,^^59^^–^^62^^,^^107^^,^^217^ The majority of reviewed studies used parametric methods for statistical data analysis. In fNIRS studies, the assumptions for parametric tests are sometimes violated (e.g., normal distribution due to small sample size); therefore, nonparametric tests are a considerable option.^218^^,^^219^ Moreover, nonparametric tests are more robust and less influenced by outliers or nonnormal distributed data^220^^–^^222^ and are recommended to use in fNIRS studies. From another point of view, in neuroscience, multiple experimental conditions (crossed) or multiple observations per condition (nested) were used.^223^^,^^224^ Furthermore, different categorical or continuous confounding variables have to be considered (e.g., gait speed, education, and gender) and/or data were unbalanced or incomplete, which makes it necessary to use advanced statistical methods.^223^^,^^225^ To account for those problems, linear mixed-effect models can be used.^10^^,^^224^^–^^226^ However, statistical methods should be chosen carefully considering the experimental design and distribution of recorded data. A further description of statistical methods for fNIRS data is given in the reviews of Tak and Ye^192^ and Kamran et al.^227^

#### 5.1.9 Markers for the assessment of cortical activation

The majority of reviewed studies used only oxyHb for the quantification of cortical activation since a change in oxyHb is assumed to be a more robust marker of changes in regional cerebral blood flow than changes in deoxyHb.^160^^,^^228^^,^^229^ However, this procedure seems questionable because neuronal activity is not just mirrored in an increase of oxyHb but also in a decrease in deoxyHb in healthy adults.^30^^,^^230^ Furthermore, an enhanced level of physiological noise is more prominent in oxyHb signals^30^ and the decrease in deoxyHb is related to an increase in BOLD contrast obtained in fMRI^231^^,^^232^, which supports the validity of the evaluation of deoxyHb changes. In pathological states, neurovascular coupling might perhaps be impaired, which results in altered concentration changes in deoxyHb during neural activity.^230^ Lindauer et al.^230^ assumed that in some pathological states, an increase in deoxyHb may reflect neural activity. Based on the mentioned assumptions, it seems favorable to report at least oxyHb as well as deoxyHb to assess task-dependent activity.^30^^,^^45^^,^^165^

### 5.2 Discussion: Main Findings

#### 5.2.1 Walking

**Fig. 3:**
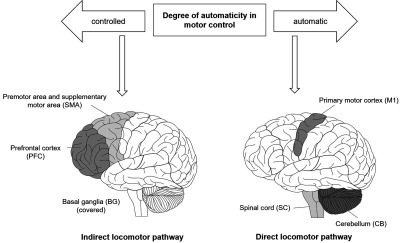
Schematic illustration of the indirect and direct locomotor pathways as a function of the degree of automaticity in motor control.

Evidence from neuroimaging studies point out that two distinct supraspinal locomotor networks are responsible for the control of walking and standing^1^^,^^233^^–^^237^ (see Fig. 3). The direct locomotor network consists of the primary motor cortex (M 1) and the cerebellar locomotor region and is potentially activated in the absence of pathologies or challenging situations.^235^ In the indirect locomotor pathway, the neuronal commands are transmitted via PFC and SMA to the basal ganglia and subthalamic as well as mesencephalic locomotor regions.^233^^–^^237^ The indirect locomotor pathway becomes activated when the automatic execution of walking is impaired (e.g., in challenging situations) and compensatory mechanisms are necessary.^44^^,^^238^ This assumption is supported by findings of our reviewed fNIRS studies, which reported more pronounced activation in prefrontal structures in (1) in adults during dual-task walking,^57^^,^^63^^,^^63^^,^^66^^,^^69^^,^^72^^–^^74^^,^^77^^,^^86^^,^^89^ (2) in adults during fast walking,^53^^,^^64^ (3) in obese persons,^87^ (4) in individuals with low gait capacity during fast walking,^53^ (5) in older adults with high level of perceived fatigue^85^ or stress,^84^ (6) in old adults with increased fall risk,^83^ and (7) in neurological patients.^58^^,^^70^^,^^71^^,^^75^^,^^80^^,^^82^^,^^90^ Remarkably, the PFC activation in neurological patients correlates with their step widths,^71^ which again (1) is associated with balance control^239^ and (2) serves as a predictor of falls.^240^ Furthermore, correlations between cortical activation and motor performance,^55^^,^^56^ especially obvious in dual-task walking conditions,^76^^,^^77^^,^^89^^,^^105^ was observed. This reinforces the important role of cortical areas in motor control. Moreover, the reduction of PFC activity after a motor-cognitive intervention program (lasting 8 weeks)^68^ perhaps originated from the shift toward a more automatic control of locomotion relying on the enhanced usage of direct locomotor pathway via M1, cerebellum, and spinal cord.^1^^,^^233^^,^^234^^,^^238^

However, premotor areas and the SMA play a role in different cognitive processes^241^^–^^243^ and were activated as a function of task difficulty in a variety of cognitive domains.^244^^–^^246^ Hence, the phenomenon of a more pronounced activation of premotor areas (as part of indirect locomotor pathway) in diseased cohorts (or during challenging motor tasks) is perhaps not fully attributable to motor task complexity but partly also to general task complexity.

However, the decrease in PFC activity in a complex visual task^105^ or difficult working memory tasks during walking^79^^,^^88^ may not be induced by the shifts in locomotor pathways but rather originate from the prioritization of task-relevant areas as consequence of the limited resources of the brain.^247^ While those three studies focused only on PFC activity, it is difficult to draw a final conclusion about potentially underlying cortical processes in other areas. Hence, to elucidate the mechanisms with respect to task prioritizations, we require further research^248^^,^^249^ including the simultaneous assessment of more cortical structures (e.g., motor areas).

For the design and monitoring of rehabilitative interventions, fNIRS could be a promising tool.^42^ For instance, the SMC activity decreases during weight-supported walking in stroke patients^59^ and could be a hint that weight supports lower task complexity.^250^ Interestingly, a verbal preadvice^67^^,^^94^ or the usage of mechanical assistance during walking^61^^,^^106^ increases central nervous load. These findings could be useful to create tailored rehabilitation programs that consider mental load as variable for workload assessment.

#### 5.2.2 Postural tasks

As pointed out for walking, neural control of posture is realized via direct or indirect pathway^251^ which are shown in Fig. 3. Our results reveal that the PFC activation is enhanced in (1) neurological patients during standing^93^ or during postural perturbations^95^^,^^102^ and (2) healthy adults during challenging balance tasks.^91^^,^^98^^,^^99^ These findings and the observations that PFC activity and SMA are associated with balance measures^95^^,^^100^^,^^102^ support the notion that indirect locomotor pathway is crucial for neuromotor processes in nonautomatized challenging situations.

Additionally, altered sensory information evoked by the execution of SOT induces a higher activation especially in STG.^92^^,^^96^ The STG is associated with (1) the control of more difficult balance tasks,^97^ (2) the integration of vestibular information,^252^^–^^254^ and (3) the spatial orientation.^255^ So far, the mentioned studies did include only young participants.^92^^,^^96^ While aging changes the contribution of somatosensory, vestibular, and visual system in balance tasks,^256^ it seems necessary to enlarge existing knowledge about cortical sensory integration processes.

## 6 Key Studies

In the following, we highlight one key study in the area of walking and balance. Those studies are of high practical relevance and cannot be performed in an fMRI since motor imagery is suggested not to be a satisfactory indicative of brain activation during motor execution.^257^

### 6.1 Walking

The usage of a smartphone during walking causes serious injuries.^258^^,^^259^ Hence, the understanding and the analysis of underlying motor control processes of walking while texting on a smartphone seems to be of high practical relevance.^260^ The investigation of smartphone usage while recording the kinematics of gait is not possible in an fMRI-scanner but could be conducted with fNIRS. In the study of Takeuchi et al.,^89^ the influence of using a smartphone while walking was investigated in healthy old and young adults. Takeuchi et al.^89^ observed that in young adults, the activation magnitude of left PFC is associated with dual-task cost (change between single- and dual-task performances) of gait acceleration and right PFC is related to the dual-task cost of the conducted cognitive smartphone task. In contrast, in the older adults middle PFC was associated with dual-task costs of step time and the activation of the left PFC is associated with dual-task costs of gait acceleration.^89^ Furthermore, younger adults have lower dual-task costs in kinematic parameters.^89^ In sum, these results point toward the effective lateralization in young adults, while in older adults more resources are needed to maintain gait performance which is in accordance with the theories of hemispheric asymmetry reduction^261^ and compensational recruitment.^262^

### 6.2 Postural Tasks

While fMRT is sensitive to motion artifacts,^18^^–^^21^ the simultaneous recording of brain activity and the quantification of kinematic parameters of gross motor skills (e.g., dynamic whole-body balance task) are impossible. Remarkably, it is assumed that to increase our knowledge about neuromotor control processes, the simultaneous assessment of brain activity and kinematic parameters is necessary.^263^ Furthermore, gross motor skills are, for example, an essential part of rehabilitative interventions (e.g., balancing on wobble board^264^^–^^266^). The study of Herold et al.^100^ used fNIRS to investigate the contribution of motor areas in online neuromotor control of balance performance on a wobble board and recorded simultaneous sway parameters via an inertial sensor. They observed (1) a pronounced activation of PrG, PoG, and SMA during balancing and (2) a strong negative correlation between the magnitude of SMA activation and sway in mediolateral direction during balancing.^100^ The results of Herold et al.^100^ allow a deeper understanding of the role of the SMA in online neuromotor control of balance movements and may be helpful to design tailored intervention programs or to monitor the intervention progress.

## 7 Conclusion

In sum, neuroimaging with the fNIRS technology seems to be a promising tool to shed light on the functioning of cortical areas in motor control. However, the absence of standardized study protocols limits the comparability among studies. Based on our findings, we deduce recommendations and potential future directions, which are shown in Table 2. Hopefully, those recommendations will lay foundations to improve the study protocols and data processing of fNIRS methodology encouraging further research to extend our existing knowledge about neuromotor control processes. This increase in knowledge might be helpful to develop tailored rehabilitation programs for clinical settings in, e.g., orthopedics and neurology.^42^ Furthermore, combining the information we can derive from fNIRS signals with kinematic parameters which are risk factors for falls^132^^,^^267^ or for cognitive decline^268^ could perhaps support a more sensitive and effective early detection of persons with a high likelihood for falls or with a high risk to develop cognitive diseases. This, in turn, may allow an early onset of therapeutic interventions, an effective monitoring of intervention programs and it would support the decision making in health care units. Those potential applications could be beneficial for patients and the resources of the health care system.

**Table 2:** Recommendations for future fNIRS studies.

Recommendations:
• Report all technical configuration details (source-detector separation, wavelengths, sampling frequency, number of measurement channels, DPF values with selection process, etc.) and design-related details (e.g., duration of task and rest phases).
• Optode placement should be based on the 10 to 20 EEG system.
• Additional measures (e.g., heart rate, blood pressure, respiration, skin conductance, etc.) should be used to monitor systematic changes.
• In order to process data, the use of bandpass filters and wavelet filters is recommended.
• DPF values should be calculated depending on age and cortex region or directly quantified via frequency- or time-domain NIRS.
• Physiological cofounders (e.g., scalp blood flow) should be reduced with the aid of PCA/ICA analyses or the usage of short separation channels.
• Baseline correction or baseline normalization should be applied.
• Averaging across channels of a ROI and trials seems to be favorable.
• The relative changes of both, oxyHb and deoxyHb, should be reported and used in the statistical analysis.

## Appendix

For further information about search strategy, cohort characteristics, study protocols, number of fNIRS channels, used wavelengths and sampling frequencies in the reviewed studies, we provide supplemental content which is available in Ref. 52, or can be requested by e-mail from the corresponding author.
